# Investigation of the Structural Heterogeneity and Corrosion Performance of the Annealed Fe-Based Metallic Glasses

**DOI:** 10.3390/ma14040929

**Published:** 2021-02-16

**Authors:** Dandan Liang, Jo-Chi Tseng, Xiaodi Liu, Yuanfei Cai, Gang Xu, Jun Shen

**Affiliations:** 1 College of Mechatronics and Control Engineering, Shenzhen University, Shenzhen 518060, China; liangdandanup@szu.edu.cn (D.L.); jochi.tseng@szu.edu.cn (J.-C.T.); xdliu2018@szu.edu.cn (X.L.); xugang@szu.edu.cn (G.X.); 2 Key Laboratory of Optoelectronic Devices and Systems of Ministry of Education and Guangdong Province, College of Optoelectronic Engineering, Shenzhen University, Shenzhen 518060, China; 3 College of Materials Science and Engineering, Tongji University, Shanghai 201804, China; yzcaiyuanfei@tongji.edu.cn

**Keywords:** metallic glass, synchrotron radiation, heterogeneity, electrochemical behavior, passive film

## Abstract

This study investigated the structural heterogeneity, mechanical property, electrochemical behavior, and passive film characteristics of Fe–Cr–Mo–W–C–B–Y metallic glasses (MGs), which were modified through annealing at different temperatures. Results showed that annealing MGs below the glass transition temperature enhanced corrosion resistance in HCl solution owing to a highly protective passive film formed, originating from the decreased free volume and the shrinkage of the first coordination shell, which was found by pair distribution function analysis. In contrast, the enlarged first coordination shell and nanoscale crystal-like clusters were identified for MGs annealed in the supercooled liquid region, which led to a destabilized passive film and thereby deteriorated corrosion resistance. This finding reveals the crucial role of structural heterogeneity in tuning the corrosion performance of MGs.

## 1. Introduction

Due to the homogenous amorphous structure (no grain boundaries, inclusions, or precipitates), metallic glasses (MGs) exhibit superior strength and hardness, excellent corrosion resistance, low cost, etc. [1,2]. However, with the continuous exploration of amorphous structure, the homogenous microstructure of MGs is found out to be heterogeneous at the nanoscale or microscale. For instance, Hirata et al. proved the local atomic order in metallic glasses by nanobeam electron diffraction combined with ab initio molecular dynamics simulation [3] and then revealed the relation between the nanoscale spatial heterogeneity and the local structure variation from icosahedron-like to tetragonal crystal-like order in MGs [4]. Wagner and Liu et al. characterized the inhomogeneous distribution of local elastic modulus and energy dissipation utilizing atomic force microscopy (AFM) [5,6], which quantified the structural heterogeneity in MGs. The high-energy X-ray or neutron diffraction could detect the spatial heterogeneity in MGs from atomic-scale to nanoscale without destroying the sample [7,8].

Recently, understanding the structural heterogeneity of MGs and its relation to the intrinsic properties is an area of interest for researchers [9]. Intensive studies focused on the relationships between the structural heterogeneity of MGs and superplasticity [10,11], thermal expansion [12], magnetoelastic effect [13], catalytic performance for dye degradation [14], electrochemical supercapacitors [15], and hydrogen generation [16]. Nevertheless, the correlation between structural heterogeneity and corrosion is still an open topic. On the one hand, the decreased free volume promotes the formation of a highly protective passive film and enhances the corrosion resistance of the relaxed MGs [17,18]. Tailleart et al. [19] reported that the structural relaxation favors the solute enrichment and passivation because it eliminates free volume and annihilates vacancies. The nanoscale elemental partitioning of MGs determines the degree of passivation [20]. On the other hand, Jindal and co-workers [21] unveiled that the structural changes are irrelevant with the improved passivity of the relaxed MGs. Moreover, the structural relaxation reduces the atomic mobility of strong passivating elements in MGs and further inhibits the formation of passive films [22]. Thus, the correlation between the structural heterogeneity and corrosion performance remains to be understood.

In this study, Fe_43_Cr_20_Mo_10_W_4_C_15_B_6_Y_2_ (at %) glassy ribbons with high glass-forming ability (GFA) and excellent corrosion resistance were examined. The structural heterogeneity, electrochemical behavior, and passive film characteristics of MGs were modified through isothermal annealing at different temperatures. The effect of annealing on the structural heterogeneity of Fe-based MGs was realized from a microscale perspective via a combination of synchrotron radiation (SR) X-ray total scattering and high-resolution transmission electron microscopy (HRTEM) techniques. In addition, the correlation between structural heterogeneity and corrosion performance of MGs was also discussed.

## 2. Materials and Methods

### 2.1. Preparation and Characterization of MGs

Master ingots with a nominal composition of Fe_43_Cr_20_Mo_10_W_4_C_15_B_6_Y_2_ (at %) were prepared by arc melting a mixture of commercial-purity Fe (99.95 wt %), Cr (99.95 wt %), Mo (99.95 wt %), W (99.95 wt %), Y (99.9 wt %), C (99.95 wt %), and B (99.9 wt %) in Ti-gettered Ar atmosphere. Approximately 40 μm thick and 1.2 mm wide metallic glasses (MGs) were produced using a single-wheel melt-spinning technique in Ar atmosphere with a wheel speed of 35 m/s.

**Figure 1:**
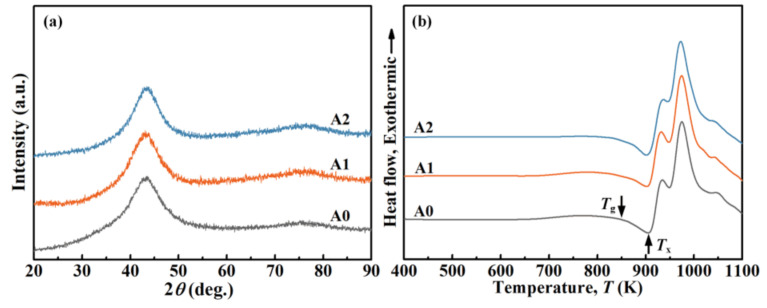
(**a**) X-ray diffractometer (XRD) patterns and (**b**) differential scanning calorimeter (DSC) curves of the initial and annealed Fe-based MGs.

The thermal stability of the melt ribbons was determined by a differential scanning calorimeter (DSC, Netzsch 404C, Waldkraiburg, Germany) with a heating rate of 20 K/min. Based on the DSC responses in Figure 1b, the glass transition temperature (*T*_g_) and the onset of crystallization temperature (*T*_x_) of the initial A0 samples were measured to be approximately 858 and 920 K, respectively. Afterward, A0 samples were heated to 0.7 *T*_g_ and *T*_g_ + 10 K for 30 min under Ar flow to obtain two independent A1 and A2 samples, respectively. Phase characterization of MGs was carried out by utilizing an X-ray diffractometer (XRD, Rigaku MiniFlex600, Austin, TX, USA) accompanied with Cu K_α_ radiation.

To exploit the structural heterogeneity of Fe-based MGs, the total scattering experiments were conducted at Beamline P02.1, DESY (PETRA III), where the high-energy X-ray of about 60 keV (wavelength ≈0.207 Å) was beneficial for a large scattering vector (*q*) range collection. Each measurement was collected with 30 s detection, and the Perkin-Elmer EN1621 2D detector (Hamburg, Germany) was used. The sample-to-detector distance was set to 243 mm, and the *q*_max_ of the total scattering data reached 24 Å^−1^. The raw 2D scattering data were integrated by DAWN [23,24]. Subsequently, the pair distribution function (PDF) was obtained using the software package PDFgetX2 to understand the local structure of the samples [25]. The *q* range was limited to 1–20 Å^−1^ in the Fourier transform because of low statistics at high scattering angles. Furthermore, the microstructure was observed via a transmission electron microscope (TEM, JEM F200, Akijima, Tokyo, Japan) with an operating voltage of 200 kV. TEM foils were prepared using a focused ion beam (FIB, FEI Scios, Hillsboro, OR, USA), and the specific FIB parameters were clarified in our previous work [26].

### 2.2. Nanoindentation Tests

The mechanical property of MGs was investigated by nanoindentation tests utilizing a triboindenter (Hysitron TI-950, Eden Prairie, MN, USA). Prior to testing, the triboindentater was calibrated with fused quartz. Nanoindentation experiments were performed in a load-control mode using a diamond Berkovich indenter. The constant loading and unloading rates were 1600 μN/s, the maximum applied load was 8000 μN, and the holding time at maximum load was 2 s in all cases. Each indentation test was repeated nine times to ensure the acceptable reliability of the data collected.

### 2.3. Electrochemical Measurements

Electrochemical measurements were performed with a three-electrode electrochemical workstation (Gamry Reference 60+) with a platinum sheet as the counter electrode, a saturated calomel electrode (SCE) as the reference electrode, and the samples as the working electrode. First, 1 mol/L HCl solution was selected as the electrolyte. Prior to the electrochemical tests, all samples were mechanically ground with 2000-grit metallographic sandpaper, cleaned with ethanol, and dried. Once the open circuit potential (OCP) was stabilized after immersing in the electrolyte for 30 min, electrochemical impedance spectroscopy (EIS) tests were conducted at the OCP with a sinusoidal amplitude of 10 mV and a frequency range of 10^5^–10^−2^ Hz. The EIS outcomes were fitted on the basis of the equivalent electrical circuit via ZSimpWin software (Version 3.30d, AMETEK Scientific Instruments, Michigan, USA). The potentiodynamic polarization was executed from −0.3 V_OCP_ to +1.2 V_SCE_ with a sweep rate of 0.5 mV/s. The samples were independently polarized at 0.5 V_SCE_ for 30 min in 1 mol/L HCl solution to obtain stable passive films. Afterward, the Mott–Schottky measurements of the formed passive films were conducted from 0 to 1.0 V_SCE_ with a measurement frequency of 10^3^ Hz and a scan rate of 10 mV/s. All electrochemical measurements were performed at room temperature and repeated thrice to guarantee data reliability.

### 2.4. XPS Analysis

The depth profiles of the passive films that formed on the surface of MG samples were examined through X-ray photoelectron spectroscopy (XPS, ESCALAB250Xi, Waltham, MA, USA) with a monochromated Al K_α_ X-ray source (*hν* = 1486.6 eV) and a controlled Ar-ion beam sputtering rate of 0.05 nm/s. The binding energy was calibrated by setting the Au *4f*_7/2_ peak of pure Au sample at a binding energy of 83.96 ± 0.02 eV. XPS responses were analyzed via CasaXPS software (Version 2.3.16, Los Angeles, CA, USA). Prior to the tests, all samples were ultrasonically cleaned with alcohol to eliminate any surface impurities.

## 3. Results and Discussion

### 3.1. Characteristics of Fe-Based MGs

The XRD patterns of the initial and annealed MG samples in Figure 1a show a similar configuration, namely, a broad halo peak around 2*θ* range of 40–50° without any crystalline diffraction peak, indicating their amorphous traits within the XRD resolution [27]. Moreover, all DSC thermograms in Figure 1b exhibit a wide supercooled region Δ*T*_x_ (*T*_x_ − *T*_g_) followed by three exothermic crystallization events. As shown in Figure 1b, the exothermic heat that occurs below *T*_g_, that is, the structural relaxation exothermic heat (Δ*H*_rel_) decreases from ≈7.51 J/g for the A0 sample to ≈6.71 J/g for the A1 sample and ≈0 J/g for the A2 sample, respectively. It is noteworthy that the difference in Δ*H*_rel_ is proportional to the amount of annihilated free volume during the isothermal annealing process [28,29,30]. Therefore, the decreased Δ*H*_rel_ corresponds to the release of free volume in the amorphous structure, especially the elimination of most of the free volume in the A2 MG. Moreover, the crystallization enthalpy (Δ*H*_cry_) of A0, A1, and A2 samples, namely, the integrity of crystallization peaks above *T_g_* [31], are measured to be ≈87.50, ≈87.84, and ≈76.56 J/g, respectively. Compared to the original A0 sample, the nearly same Δ*H*_cry_ of A1 sample suggests the unoccurring crystallization during annealing below *T*_g_, whilst the slightly reduced Δ*H*_cry_ of A2 sample indicates the sporadic crystallization during annealing in the supercooled liquid region. From the perspective of potential energy landscape (PEL), many local potential energy minima (also called inherent structures or ISs) exist in amorphous materials, and they represent the metastable states [32,33,34]. Annealing below *T*_g_ induces hopping between contiguous ISs, thus modifying the glassy structure with different degrees of disorder in A1 MG [35]. However, annealing MG in the supercooled liquid region can reduce the energy barrier between the glassy phase and crystalline phase, resulting in the heterogeneous nucleation sites in A2 MG [36]. Whereafter, high-resolution characterizations were taken to further understand the effect of annealing on the structural heterogeneity of MGs.

**Figure 2:**
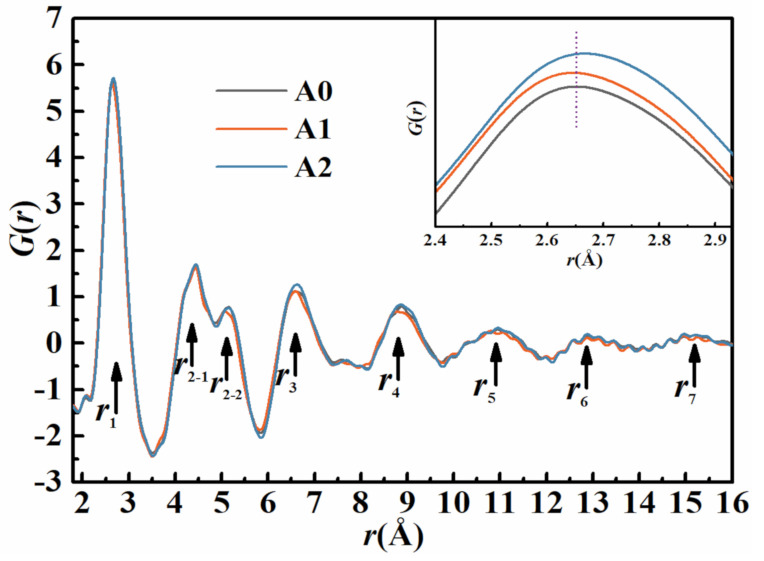
Pair distribution functions of Fe-based metallic glasses (MGs).

SR X-ray total scatting experiments were performed to exploit the local atomic structure information of Fe-based MGs. PDF analysis is used to calculate *G*(*r*), which is a pair correlation that represents the probability of finding atoms as a function of distance *r* from an average center atom [37]. In Figure 2, all *G*(*r*) patterns show strong oscillation spanning from 2 to 16 Å, reflecting the existence of short-range order (SRO) and medium-range order (MRO) in the atomic arrangement of all tested samples. The fluctuating pattern and the splitting of the second peaks reveal the typical amorphous traits [38]. The radius of the first coordination shell, given in *r*_1_, of the initial A0 and annealed A1 samples are determined to be 2.65 and 2.63 Å, respectively. An opposite observation is shown for the annealed A2 sample. The shift of *r*_1_ from 2.65 to 2.67 Å indicates a larger first coordination shell and lower atomic packing density in the local structure of A2 sample. The enhanced intensities of the *r*_3_ and *r*_4_ peaks signify the increased amount of MROs for MGs annealed in the supercooled liquid region.

**Figure 3:**
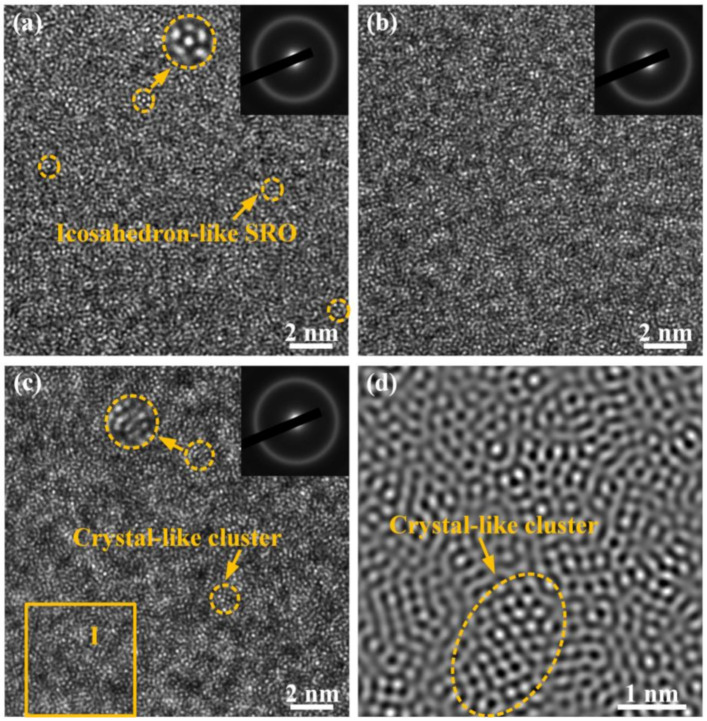
HRTEM images and selected area electron diffraction (SAED) patterns of (**a**) the initial and annealed MGs at (**b**) 0.7 *T*_g_ and (**c**) *T*_g_ + 10 K, and (**d**) inverse fast Fourier Transform (IFFT) pattern of Region I.

To give a solid vision in the structure–property relationship, not only the PDF analysis giving as averaging information but also the HRTEM and the selected area electron diffraction (SAED) were applied to characterize locally atomic-scale structure changes in MGs. As shown in Figure 3a–c, the bright halos in the SAED patterns demonstrate the amorphous structure in all three samples. The HRTEM image of A0 sample in Figure 3a depicts a maze configuration without any crystalline fringes. The existence of abundant icosahedron-like SROs with five-fold symmetry is the structural origin of its superior GFA [39]. For the A1 sample, annealing at 0.7 *T*_g_ assists the release of free volume, the elimination of “liquid-like” regions, and the generation of numerous “solid-like” regions in the amorphous structure [40]. As a result, the atomic arrangement of A1 MG is more compact, which is in accordance with DSC and PDF analysis. However, for MG annealed at *T*_g_ + 10 K, namely the A2 sample, several nanoscale crystal-like clusters with an ordered atomic arrangement are observed (Figure 3c). Local straight lattice fringes are illustrated in the inverse fast Fourier Transform (IFFT) pattern of Region I, as depicted in Figure 3d. In addition, the enhanced intensities of *r*_3_ and *r*_4_ peaks in G(r) pattern illustrate the increased amount of MROs, which correspond to the nanoscale crystal-like clusters in the HRTEM image for A2 MG. These crystal-like clusters can be regarded as nuclei seeds and trigger an “avalanche-like nucleation” during the further crystallization of MGs [39]. However, the SAED pattern in Figure 3c only exhibits one bright halo without diffraction spots, which is probably because the content and size of crystal-like clusters in the A2 sample are too small, as indicated by the DSC, PDF, and TEM results. It is noteworthy that these distinct microstructures in three samples are considered as the crucial factors in tuning the mechanical and corrosion properties of MGs.

### 3.2. Mechanical Property of Fe-Based MGs

**Figure 4:**
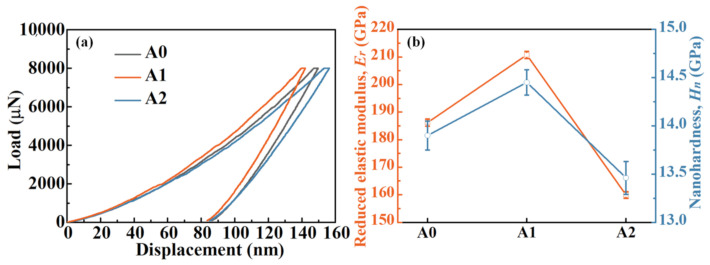
(**a**) Nanoindentation load–displacement plots and (**b**) the calculated *E*_r_ and *H_n_* of Fe-based MGs.

The typical nanoindentation load–displacement curves of Fe-based MGs are presented in Figure 4a. Under the same load and loading rate, the variation of the maximum contact depth is in the following tendency: A2 > A0 > A1, indicating the hardest characteristic of A1 MG. Furthermore, the variation of the slope of the unloading segments shows the opposite tendency, which suggests the highest stiffness of A1 MG. The specific reduced elastic modulus (*E*_r_) and nano-hardness (*H_n_*) can be calculated on the basis of the Oliver and Pharr method [41]. For the A0 sample, *E*_r_ is 185.72 GPa and *H_n_* is 13.75 GPa. As expected, *E*_r_ and *H_n_* of the A1 sample increase to 210.72 and 14.45 GPa, respectively. The increase in modulus usually indicates a decrease in the atomic distance [42]. As indicated by the aforementioned analyses, the released free volume in A1 MG results in a more compact atomic arrangement and thereby a higher *E*_r_. The initiation of shear bands is supposed to be more difficult in local sites with less free volume. Thus, the sequential shear bands in A1 MG require higher stresses to form, which leads to higher hardness [43]. Interestingly, after annealing MG above *T_g_*, *E*_r_ and *H_n_* of the A2 sample decrease to 160.24 and 13.61 GPa, respectively. This phenomenon can be explained by the enlarged atomic distance in local sites and the precipitation of nanoscale crystal-like clusters in A2 MG, as shown in Figure 2 and Figure 3. These medium-range ordering (MRO) clusters disturb the relatively disordered atomic arrangement [44,45] and might bring about the enlarged atomic distance in local sites, thereby reducing the modulus and hardness of A2 MG. Therefore, the variation in modulus is highly consistent with the PDF analysis.

### 3.3. Electrochemical Measurements of MGs

**Figure 5:**
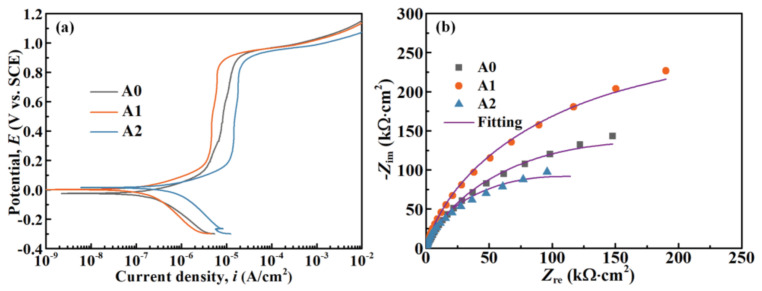
(**a**) Potentiodynamic polarization and (**b**) Nyquist curves of the initial and annealed Fe-based MGs.

**Figure 6:**
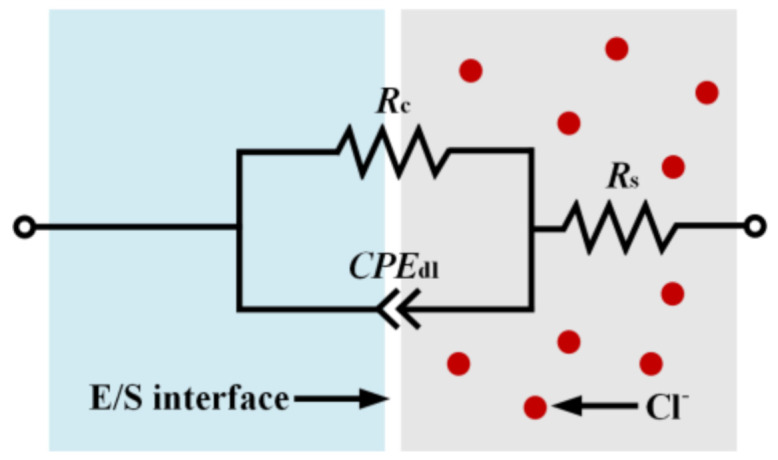
Equivalent electrical circuit model utilized to simulate the electrochemical impedance spectroscopy (EIS) spectra. Electrode/solution (E/S) interface represents the electrode–solution interface.

All potentiodynamic polarization curves in Figure 5a show a wide current density plateau and a highly positive passive–active transition potential, which indicates the superior passivity and pitting resistance of MGs. As a result of the high chemical stability of Cr-, Mo-, and W-oxides in HCl solution, a passive barrier film could be spontaneously formed, resulting in a low passive current density (*i*_pass_) with a magnitude of 10^−5^–10^−6^ A/cm^−2^. Moreover, all Nyquist plots in Figure 5b depict only one capacitive loop, which implies the single time-constant representation of the impedance behavior at the electrode/solution (E/S) interface [46]. The equivalent electrical circuit in Figure 6 matches well with the EIS spectra. Herein, *R*_s_, *R*_c_, and *CPE*_dl_ represent the solution resistance, charge transfer resistance, and deviation from a non-ideal capacitor, respectively. The calculated electrochemical parameters are summarized in Table 1. For the A1 sample, the lowest *i*_pass_ and highest pitting potential *E*_pit_, which are 4.62 µA/cm^2^ and 938.87 mV, respectively, indicate that the passive film formed on the surface of the A1 sample displays the best barrier characteristic among all others. Contrary to the A1 sample, the highest *i*_pass_ (13.67 µA/cm^2^) and the lowest *E*_pit_ (886.00 mV) are detected for the A2 sample, revealing the weakened protectiveness of the passive film formed on its surface. Moreover, the fitted *R*_c_ values of the A0, A1, and A2 samples are 300.32, 458.75, and 202.46 kΩ·cm^2^, respectively. High *R*_c_ refers to superior passive film stability and excellent corrosion resistance [47]. Hence, the fitted EIS outcomes are highly consistent with the potentiodynamic polarization results. In brief, annealing MG at 0.7 *T*_g_ leads to a decreased *i*_pass_, elevated *R*_c_, and increased *E*_pit_, indicating the enhanced corrosion resistance. Whereas, annealing MG at *T*_g_ + 10 K results in an increased *i*_pass_, declined *R*_c_, and decreased *E*_pit_, suggesting the deteriorated corrosion resistance.

### 3.4. Semiconducting Properties of Passive Films

**Figure 7:**
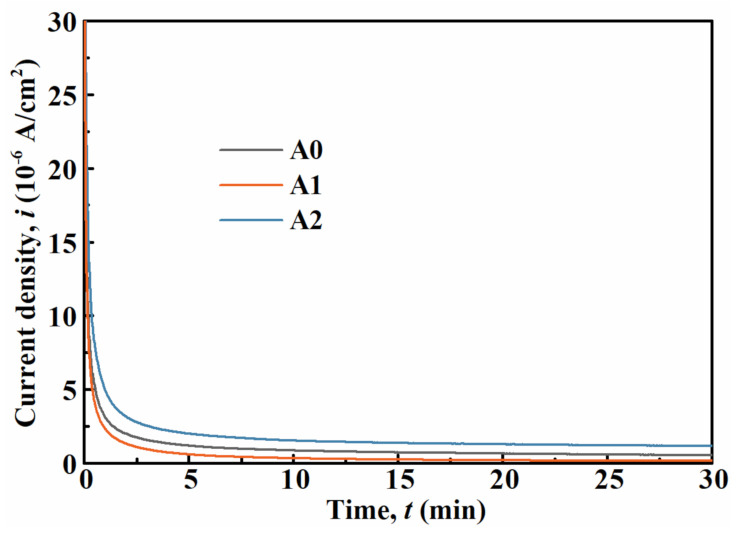
Potentiostatic polarization curves of MGs.

As shown in Figure 5a, all MGs exhibit superior passivation behaviors in the potential range of 0.2–0.8 V_SCE_. These MGs were polarized at 0.5 V_SCE_ for 30 min to obtain passive oxide films. The current density (*i*)–time (*t*) responses in Figure 7 present that *i* begins to drop sharply and eventually reaches a steady-state with the increase in *t*, suggesting the formation of passive films on the surfaces of MGs [48]. Endowed with the minimum *i* at the steady-state, A1 MG exhibits its superior corrosion resistance to the others, which is highly consistent with the potentiodynamic polarization and EIS results.

The corrosion resistance of metallic glasses is highly related to their passive film characteristics, such as the semiconducting property, chemical composition, and thickness. Given the imperfection of passive oxide films from crystallographic aspects, these films are generally identified as an *n*-type semiconductor for donor defects or a *p*-type semiconductor for acceptor defects [49]. The semiconducting properties of passive films are evaluated by the Mott–Schottky approaches, which represent the measured electrode capacitance as a function of the electrode potential. Generally, the electrode capacitance *C*_f_ is expressed as follows:

(1) $$C_{f}{}^{-2}=C_{\mathrm{SC}}{}^{-2}+C_{H}{}^{-2}+2C_{\mathrm{SC}}{}^{-1}C_{H}{}^{-1}$$

where *C*_SC_ and *C*_H_ stand for the capacitance of the space charge layer and Helmholtz layer, respectively. The availability of Mott–Schottky analysis hinges on the assumption that *C*_SC_ is much smaller than *C*_H_. Under the test frequency of 10^3^ Hz, the contribution of $2C_{\mathrm{SC}}{}^{-1}C_{H}{}^{-1}$ and $C_{H}{}^{-2}$ in Equation (1) is negligible; thus, the measured *C*_f_ equals to *C*_SC_. For *n*-type semiconductors, *C*_SC_ is expressed in Equation (2) [50]:

(2) $$C_{\mathrm{SC}}{}^{-2}=\frac{2}{\varepsilon_{0}\varepsilon_{r}N_{D}e}\left(E-E_{\mathrm{FB}}-\frac{kT}{e}\right)$$

where *ε_0_* is the vacuum permittivity; *ε_r_* is the dielectric constant of the passive film (*ε_r_* = 15.6 [51]); *e* is the electron charge; *E* is the applied potential; *E_FB_* is the flat band potential; *k* is the Boltzmann constant; *T* is the temperature in Kelvin; and *N_D_* is the donor density, which is determined in Equation (3):

(3) $$N_{D}=\frac{2}{\varepsilon_{0}\varepsilon_{r}e}{\left(\frac{dC^{-2}}{dE}\right)}^{-1}$$

**Figure 8:**
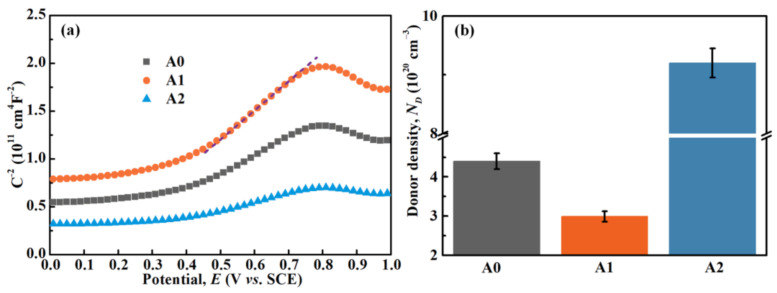
(**a**) Mott–Schottky plots and (**b**) the calculated *N*_D_ of the passive films that formed on MGs.

Mott–Schottky responses of the passive films formed on MGs are shown in Figure 8a. All three curves exhibit similar shapes with positive linear regions in the potential range of 0.4–0.7 V_SCE_, illustrating that the passive films represent the *n*-type semiconductor and comprise the massive donor defects in this potential range [52]. As shown in Figure 8b, the calculated *N*_D_ values of passive films formed on the A0, A1, and A2 samples are approximately 4.40 × 10^19^, 2.99 × 10^19^, and 9.20 × 10^19^ cm^−3^, respectively. Lower *N*_D_ indicates lower conductivity and better protective characteristics for passive films [53]. Thus, by reviewing the aforementioned *N_D_* values, the passive film formed on A1 MG is relatively protective, whilst that formed on A2 MG is defective.

### 3.5. XPS Depth Profiling Analysis

**Figure 9:**
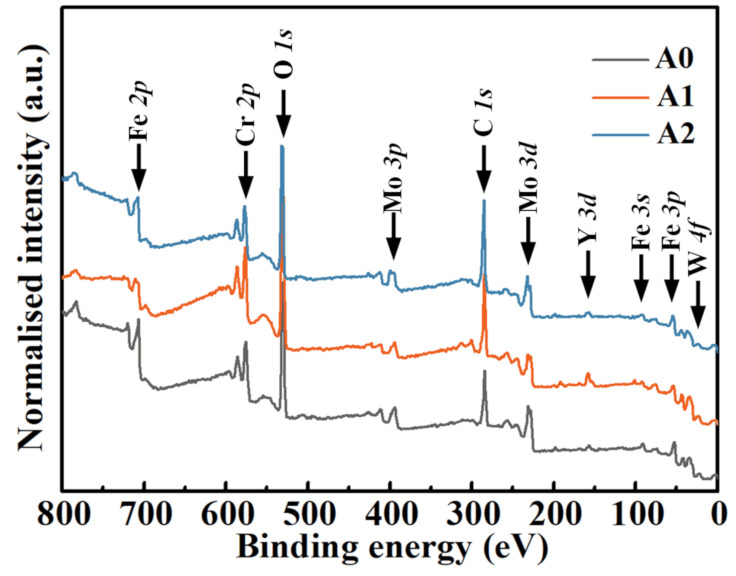
XPS survey spectra of the passive films without sputtering that formed on MGs.

**Figure 10:**
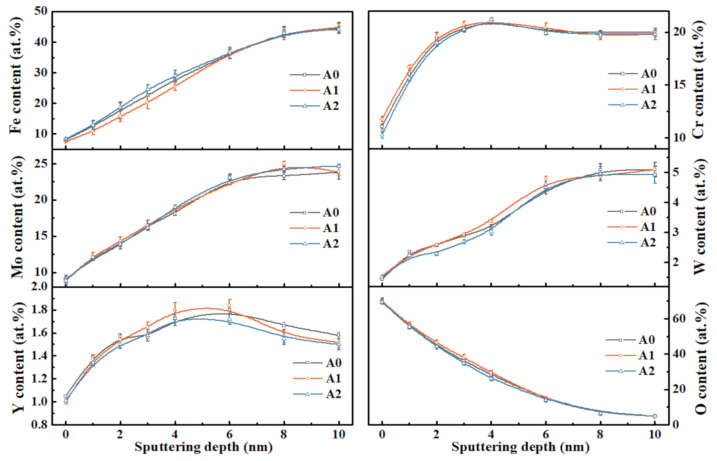
Depth profiles of elemental concentrations of the passive films that formed on the initial and annealed MGs.

XPS on analyzing the passive films of MGs (Figure 9) indicates the presence of Fe, Cr, Mo, W, Y, and O elements. The C element was excluded because it was considered as a contaminant during the measurement. The relative atomic concentrations of each element in passive films, calculated from the integrity of the peak area, are given in Figure 10. Apparently, the cationic Fe, Cr, Mo, W, and Y elements and the anionic O element in the passive films are diffused from the metal matrix and the electrolyte side, respectively. Among the three samples, the Cr concentration of the passive film surface that formed on A0, A1, and A2 samples is approximately 11.1 at %, 11.8 at %, and 10.3 at %, which is in a trend of A1 > A0 > A2. The surface enrichment of the Cr element could accelerate the formation of a passive film with greater resistance to the attack of Cl^–^ ions in acid media [54]. Therefore, a better corrosion-resistant performance can be expected for the A1 sample. Interestingly, the thicknesses of the passive films, which was considered as the thickness of oxide layers where the oxygen content is approximately half that of the initial surface [51,55], have the same trend as the concentration of surface Cr for the three samples. The calculated thicknesses, marked in a dashed line, are approximately 3.17, 3.33, and 2.99 nm for A0, A1, and A2 MGs, respectively. This result demonstrates that annealing MG at 0.7*T*_g_ thickens the passive film and improves its protectiveness. Instead, annealing MG at *T*_g_ + 10 K has an opposite effect on the passive film.

Combined with the Mott–Schottky and XPS results, these passive film characteristics effectively explain the electrochemical behaviors of MGs in Figure 5. With a decreased *N*_D_, higher concentration of surface Cr, and increased passive film thickness, a better passivity and excellent corrosion resistance are illustrated, suggesting a trend of A1 > A0 > A2 for Fe-based MGs. Therefore, the MGs annealed at different temperatures across the *T*_g_ region may give a distinct response to its corrosion resistance led by the multi-effects on the formation of a stable and protective passive film.

### 3.6. Effect of Annealing Temperature on the Structural Heterogeneity of MGs

According to the PEL theory, annealing MGs at different temperatures can readily obtain glassy structures with various energetic states. As shown in Figure 1b, the Δ*H*_rel_ value in DSC thermograms decreases from ≈7.51 to ≈6.71 J/g for MG annealed at 0.7 *T*_g_, which could be explained by the active local atomic motions rather than the global atomic motion. The local atomic rearrangement through short-range diffusion or cooperative atomic motion is triggered by the release of the excess enthalpy [56]. As a consequence, the free volume in the amorphous structure reduces, and the atoms pack more compactly for A1 MG [57,58,59]. This structural change may lead to the loss of active sites that respond to the external corrosion attack.

On the increasing temperature, the thermal vibration energy becomes larger than the energy barrier, and it can lead to thermal activations for atom rearrangement in MGs [60]. When the annealing temperature reaches *T*_g_, the large size atomic diffusion notably promotes the atomic transport [61]. Some fast degrees of freedom frozen in the deep glassy state are reactivated. The enlarged first coordination shell indicates that the atomic packing in the nearest neighbors becomes looser and there is more free space to move in three dimensions. Hence, annealing MG at Tg + 10 K generates larger-sized MRO clusters, as shown in the TEM images. This process is related to an incipient crystallization as the thermal activation coincides with a marked increased medium-range ordering and a dilatation of the third shell [8]. This dilatation is also evidenced by the increased *r*_3_ and *r*_4_ peaks in PDF analysis.

### 3.7. Correlation Between the Structural Heterogeneity and Corrosion Performance of MGs

Mainly, the structural heterogeneity, considering the atom packing density and the presence of a crystal-like cluster, is strongly correlated with the performance of electrochemical corrosion and the characteristics of the formed passive film. For the A1 sample, annealing MG at 0.7 *T*_g_ leads to a reduction in free volume, which in turn yields a more compact structure between atoms and decreases the chemical potential. In this case, the atoms are supposed to lie on a lower energy joint in the crystal field that makes them become less electrochemically active and cannot be easily removed during dissolution [62]. Moreover, the atomic densification favors the compactness and protectiveness of the formed surface passive films. As a result, the formation of a stable and protective passive film is favorable, and it can effectively prevent the absorption/penetration of Cl^–^ ions and other chemical exchanges on the surface of MGs, leading to a superior corrosion resistance property [22]. However, annealing MG above *T*_g_ generates some larger-sized MRO clusters. As shown in Figure 3c,d, such nanoscale crystal-like clusters were embedded in the amorphous matrix. In addition, the structural heterogeneity induces micro-electrochemical cells around the boundary of the crystal-like clusters and the amorphous matrix [63]. These cells in passive films are expected to be terminal points for the permeation of Cl^−^ ions and act as preferential sites for pitting initiation and propagation [64,65]. From a structural viewpoint, the corrosion generally originates from a loose-packing structure and the presence of defects. The loose atomic packing, as shown by PDF analysis, leaves more free space for the absorption/penetration of Cl^−^ ions. Thereby, both these distinct microstructures accelerate the corrosion process of MG.

## 4. Conclusions

In conclusion, the effect of annealing on the structural heterogeneity of Fe-Cr-Mo-W-C-B-Y MGs and its correlation with the electrochemical behavior and passive film characteristic were investigated. Annealing MG at 0.7 *T*_g_ for 30 min led to the reduced free volume and the shrinkage of the first coordination shell in the local atomic structure, which could improve the passivity and corrosion resistance, indicating from the decreased *i*_pass_, elevated *E*_pit_, and increased *R*_c_. The reason suggested was that such microstructures favored the formation of more compact and protective passive films. In contrast, MG annealed at *T*_g_ + 10 K for 30 min exhibited a distinct microstructure, considering the enlarged first coordination shell and the presence of nanoscale crystal-like clusters, which deteriorated the protectiveness of the passive film and thereby decreased the corrosion resistance. Departing from the different annealing temperatures across the *T*_g_ point for Fe-based MGs, this study successfully determined the effect of annealing on the structural heterogeneity and established its correlation with corrosion performance of Fe-based MGs. The importance of structural heterogeneity is giving new insight into understanding the corrosion mechanism of MGs.

## Figures and Tables

**Table 1:** Electrochemical parameters of the initial and annealed Fe-based MGs.

Sample	*i*_corr_ (µA/cm^2^)	*E*_corr_ (mV)	*i*_pass_ (µA/cm^2^)	*E*_pit_ (mV)	*R*_c_ (kΩ·cm^2^)
A0	0.21 ± 0.12	−25.64 ± 17.62	8.45 ± 0.31	923.01 ± 3.32	300.32 ± 12.74
A1	0.34 ± 0.15	−1.82 ± 2.56	4.62 ± 0.73	938.87 ± 0.16	458.75 ± 14.78
A2	0.54 ± 0.11	30.62 ± 14.57	13.67 ± 2.94	886.00 ± 15.24	202.46 ± 10.87

## Data Availability

The raw/processed data required to reproduce these findings cannot be shared at this time as the data also forms part of an ongoing study.
